# Domain Generalization of Histopathology Foundation Models in Multicenter, Multi-Scanner Cohorts: A Comparative Benchmark

**DOI:** 10.3390/jimaging12080381

**Published:** 2026-08-13

**Authors:** Hafsa Akebli, Vincenzo Della Mea

**Affiliations:** Department of Mathematics, Computer Science and Physics, University of Udine, 33100 Udine, Italy; vincenzo.dellamea@uniud.it

**Keywords:** domain generalization, domain shift, multi-source data, computational pathology, foundation models

## Abstract

Histopathology foundation models (FMs) have become widely used as patch-level feature extractors in computational pathology (CPath), where domain shift is a central challenge, yet their generalization ability across acquisition centers and scanning platforms remains insufficiently studied. In this work, we evaluate the domain generalization of ten state-of-the-art FMs on two multi-source datasets with different supervision settings: SemiCOL, a colorectal cancer cohort of 499 whole-slide images (WSIs) for weakly labeled slide-level binary tumor classification, and BEETLE, a breast cancer cohort of 583 WSIs for patch-level four-class tissue classification. In an ablation-style setting, FMs are used as patch-level feature extractors, with patch embeddings mean-pooled into slide-level representations for SemiCOL, and a lightweight multi-layer perceptron trained for slide-level and patch-level classification on SemiCOL and BEETLE, respectively. To test FM domain generalization, we use three evaluation protocols: a Baseline source-mixed 5-fold cross-validation (CV) and two leave-source-out CV settings that assess cross-center and cross-scanner performance. On SemiCOL, all FMs achieve near-saturated performance, indicating stable performance under acquisition-source domain shift for slide-level Tumor vs. Benign classification. In contrast, BEETLE reveals clear generalization gaps, with scanner-induced domain shift more challenging than center-induced domain shift, and class-wise results showing that performance losses concentrate in epithelial discrimination. Overall, Virchow2 shows the strongest robustness across all evaluated protocols. These findings show that standard source-mixed CV can overestimate domain generalization across centers and scanners, and that FM choice matters in multi-source cohorts, especially for more challenging CPath tasks, where cross-domain failures are more visible.

## 1. Introduction

Whole-Slide Images (WSIs) are the core input of Computational Pathology (CPath), providing a slide-scale view of tissue morphology for computational analysis and clinical decision-making. They support tasks such as tumor detection and subtyping, grading and staging, and prediction of molecular alterations, prognosis, and treatment response, among other CPath applications [1,2]. Over the past years, the advent of digital pathology and the growing availability of WSIs have facilitated the emergence of histopathology Foundation Models (FMs), which serve as general-purpose visual encoders of tissue patches and support a wide range of CPath tasks [3].

Histopathology FMs have rapidly expanded in recent years, driven by advances in Self-Supervised Learning (SSL) approaches, model architectures, and the increasing scale of digital pathology datasets. Several state-of-the-art histopathology FMs rely on Vision Transformer (ViT) backbones pretrained with DINOv2-based SSL [4], an extension of iBOT [5] that introduces pretraining components designed for scaling data and model capacity. These FMs are pretrained on datasets ranging from thousands to millions of WSIs [6], combining public datasets such as The Cancer Genome Atlas (TCGA) [7] with in-house data. The largest models reach up to one billion parameters [8,9]. Pathology FMs are now evaluated in an increasing number of benchmark studies to quantify their strengths and limitations across tasks, organs, and evaluation settings, helping researchers select appropriate models for a given application. Existing benchmarks vary in scope, with task-focused evaluations such as ovarian carcinoma subtype classification [10], microsatellite instability prediction in colorectal cancer [11], Gleason grading in prostate cancer [12], and skin cancer subtyping [13]. Broader studies compare many FMs across multiple organs and CPath tasks, including disease detection and prediction of biomarkers and prognostic outcomes [14,15], and assess how FM performance is affected by different adaptation strategies, including fine-tuning and few-shot learning [16].

In CPath, domain shift remains a central challenge for model generalizability. Variations in tissue preparation techniques and staining procedures, together with scanner-induced differences during digitization, can alter color statistics and local texture even when the underlying tissue morphology is comparable. Such domain shift can reduce performance when models are applied outside the acquisition conditions encountered during development [17,18,19]. This is particularly relevant for histopathology FMs, which are pretrained on WSIs acquired under specific laboratory and scanner conditions and then used as feature extractors on external cohorts from other medical centers, digitized with different scanners. Recent work has examined cross-center generalization of pathology FMs and proposed task-specific adaptation to reduce center signatures and demographic bias [20]. Another study benchmarked FMs on a multi-scanner dataset and proposed a contrastive loss during task-specific fine-tuning to improve cross-scanner generalization for EGFR mutation prediction in lung cancer [21]. Therefore, systematic evaluation of FM domain generalization across acquisition centers and scanners remains necessary.

In this work, we evaluate the domain generalization of ten state-of-the-art histopathology FMs on two multicenter, multi-scanner public challenge datasets, using 499 WSIs from SemiCOL for slide-level binary tumor classification in colorectal cancer, and 583 WSIs from BEETLE for patch-level four-class tissue classification in breast cancer. We follow an ablation study setting where FMs are used as patch-level feature extractors. These patch embeddings are mean-pooled into a slide-level representation for weakly labeled Tumor vs. Benign classification on SemiCOL, while for BEETLE, the patch embeddings are classified directly at the patch-level; in both cases, classification is performed using a Multi-Layer Perceptron (MLP) classifier. We then evaluate the FMs using three protocols: a standard source-mixed 5-fold cross-validation (CV) Baseline and center-held-out and scanner-held-out CV, where one acquisition source is held out for testing and the remaining sources are used for training. We quantify performance changes relative to Baseline for both tasks, using overall metrics and class-wise F1-scores to assess FM robustness to center-induced and scanner-induced domain shift. The main contributions of this work are as follows:

- A benchmark of the domain generalization of ten state-of-the-art histopathology FMs on two public multicenter, multi-scanner datasets with complementary task formulations: slide-level binary tumor classification on SemiCOL and patch-level four-class tissue classification on BEETLE.
- A systematic comparison of the evaluated FMs under a standard source-mixed 5-fold CV Baseline and center-held-out and scanner-held-out protocols within a common benchmarking pipeline to quantify performance changes under center- and scanner-induced domain shift.
- An overall and class-wise characterization of acquisition-source generalization gaps, showing that source-mixed CV can overestimate generalization across acquisition sources, that scanner shift is more challenging than center shift on BEETLE, and that performance degradation is concentrated in fine-grained epithelial discrimination, with Virchow2 emerging as the most robust model overall.

The paper is organized as follows: Section 2 describes the datasets and benchmarking pipeline; Section 3 presents the implementation details and experimental results; Section 4 discusses the findings; Section 5 outlines the limitations; and Section 6 concludes the paper.

## 2. Materials and Methods

### 2.1. Datasets

We use two multicentric, multi-scanner public challenge datasets, SemiCOL and BEETLE, for slide-level tumor status classification in colorectal cancer and patch-level tissue classification in breast cancer, respectively. To the best of our knowledge, SemiCOL was not used in the pretraining of any of the evaluated FMs, whereas BEETLE may partially overlap with pretraining data through its TCGA subset.

The SemiCOL dataset is derived from a public colorectal cancer challenge dataset [22]. In this study, we use the subset of WSIs with slide-level tumor status labels, which constitutes the majority of the challenge data and captures its multi-source acquisition setup. Table 1 summarizes the SemiCOL data used in this study. The dataset comprises 499 WSIs, of which 250 are labeled as tumor and 249 as benign, acquired at approximately 0.5μm/pixel (20×) across four medical centers in different countries and digitized using three commercial scanner models. At one center, the same slides were scanned using two different scanners, resulting in two acquisition subsets, DSW2A and DSW2B. Consequently, the 499 WSIs correspond to 399 unique patients.

**Figure 1:**
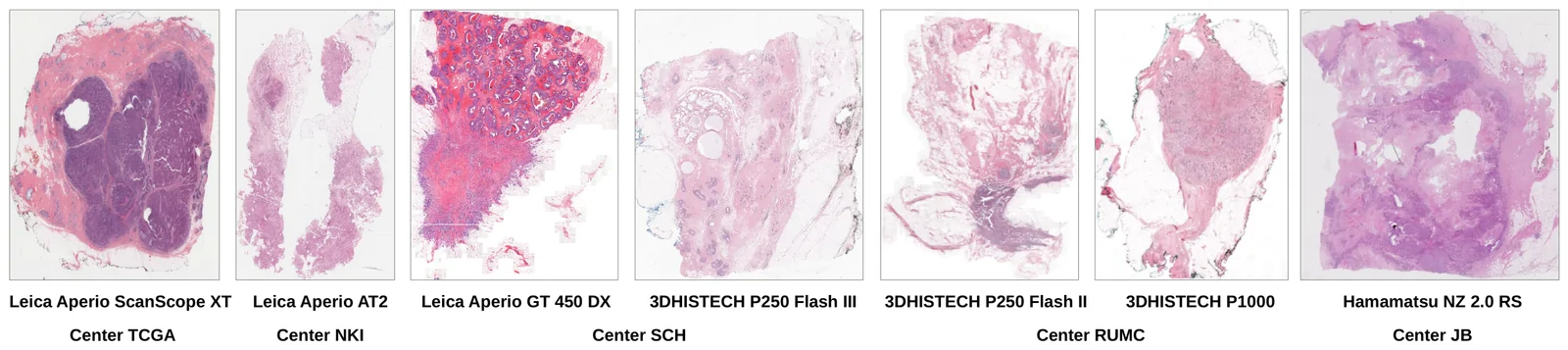
Representative BEETLE WSIs from each scanner, illustrating scanner-related appearance variability.

BEETLE is a multicenter, multi-scanner Hematoxylin and Eosin (H&E) histopathology dataset for breast cancer tissue segmentation [23]. It comprises 587 WSIs from 527 patients, all scanned at 0.5μm/pixel (20×). As summarized in Table 2, which reports the distribution of WSIs across centers and scanners in the original dataset prior to patch extraction and filtering, the slides come from five medical centers and were digitized using seven scanner models. These include one Hamamatsu NanoZoomer (NZ) scanner (Hamamatsu Photonics K.K., Hamamatsu City, Japan), three Leica Biosystems Aperio models (ScanScope XT, AT2, GT 450 DX), (Leica Biosystems Imaging, Inc., Vista, CA, USA), and three 3DHISTECH Pannoramic (P) models (P250 Flash III, P250 Flash II, P1000) (3DHISTECH Kft., Budapest, Hungary), providing multiple scanner versions for each manufacturer. The dataset provides pixel-level tissue annotations for four classes defined as Invasive epithelium, Non-Invasive epithelium, Necrosis, and Other. In this work, BEETLE is used for a patch-level tissue classification task. Figure 1 shows one representative WSI per scanner to illustrate scanner-related variation in color and visual appearance.

### 2.2. Benchmarking Pipeline

**Figure 2:**
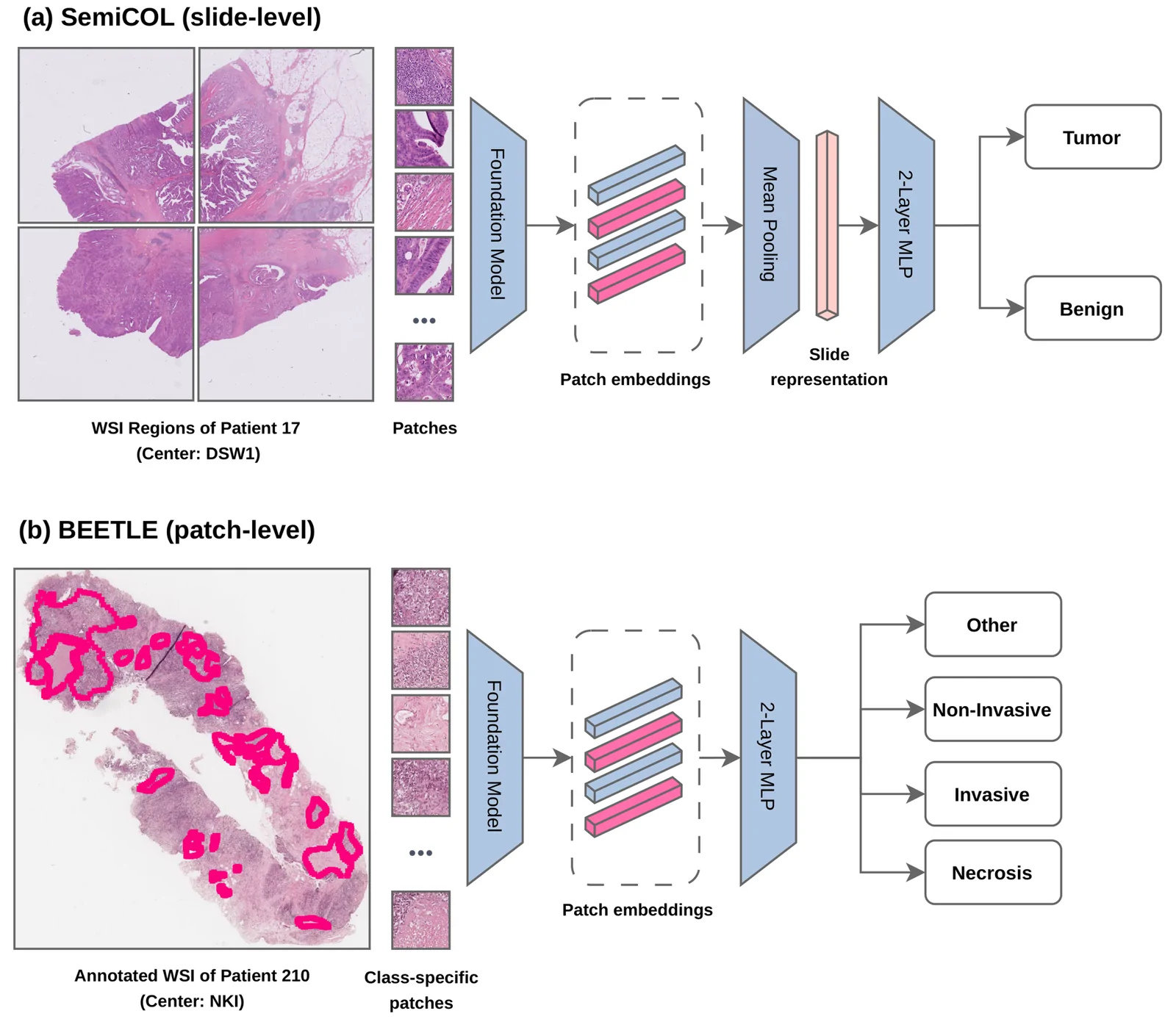
Overview of the benchmarking pipeline used to evaluate histopathology FMs on two datasets under two task formulations. (**a**) SemiCOL slide-level binary classification pipeline: WSIs are tiled into patches, FM embeddings are mean-pooled into a slide representation, and a classifier predicts Tumor vs. Benign. (**b**) BEETLE patch-level four-class tissue classification pipeline: pixel-level annotations define class-specific patches, and FM embeddings are used for four-class patch-level tissue classification.

In this subsection, we present the benchmarking pipeline used to evaluate histopathology FMs on two multi-source datasets with complementary supervision settings (Figure 2). The pipeline replicates established histopathology analysis approaches under different supervision settings. The SemiCOL pipeline formulates the problem as slide-level classification. Each WSI is tiled into patches, patch features are extracted using the FMs and aggregated into a single slide embedding for prediction. The BEETLE pipeline uses pixel-level tissue annotations to extract class-labeled patches, from which FM patch features are computed for patch-level four-class classification. The following subsections describe the dataset-specific patch extraction and quality control procedures, the set of histopathology FMs benchmarked in this study together with their main pretraining characteristics, and the evaluation protocols used to assess Baseline performance and cross-center and cross-scanner generalizability.

#### 2.2.1. Patch Extraction and Quality Control

Patch extraction was performed from the WSIs using dataset-specific strategies reflecting the available supervision. A common patch size of 512×512 pixels was used for both datasets to maintain a consistent extraction scale across tasks and FMs, while providing a practical balance between local morphological context and fine-grained tissue detail.

For the SemiCOL dataset, all WSIs were processed at an effective magnification of 20× and tiled into non-overlapping 512×512 patches, following commonly adopted tiling strategies in WSI analysis. Background regions were removed using tissue masking based on Otsu thresholding, and only patches with sufficient tissue content were retained. Out-of-focus patches were then excluded using the variance of the Laplacian, and the lowest 20% of patches according to this score were discarded.

For the BEETLE dataset, pixel-level annotation masks were used to assign patch labels. This patch-level formulation is a common approach in computational pathology, where pixel-wise annotations are aggregated into labels that match the input representation of histopathology FMs while preserving the dominant tissue semantics within each region. Each WSI was tiled at the native resolution into 512×512 patches using an overlapping sliding window with a stride of 256 pixels to ensure adequate coverage of small tissue regions that may be missed by a non-overlapping grid, and the corresponding mask regions were used to compute class pixel fractions for each patch. Each patch was assigned the label of its majority class, defined as the class covering more than 50% of its area, and patches without a dominant class were discarded to ensure a consistent conversion from pixel-level annotations to patch-level labels. Following patch extraction, the same quality control applied to the SemiCOL dataset was used to remove out-of-focus patches. For four WSIs, no patches satisfied the dominant-area criterion after patch extraction and filtering (two slides from RUMC and two slides from NKI); therefore, patches were extracted from 583 of the 587 WSIs. These 583 WSIs corresponded to 523 patients, with one to five WSIs per patient. Furthermore, for the SCH cohort scanned with the Leica Aperio GT 450 DX, no patches were assigned to the Invasive epithelium class, as this class is not present in the corresponding annotation masks. Table 3 reports the resulting distribution of extracted patches across tissue classes, stratified by center and scanner. The same patch selection and quality-control criteria were applied identically to the training and evaluation data.

#### 2.2.2. Foundation Model Feature Extraction

We benchmarked a set of ten state-of-the-art histopathology FMs for patch-level feature extraction. The selection prioritized recent and publicly available histopathology FMs while ensuring diversity in backbone architectures, pretraining strategies, model scales, and pretraining data sources. An overview of the selected models and their pretraining configurations is provided in Table 4. When multiple versions of the same model family were available, we retained the most recent variant (e.g., UNI2-h instead of UNI, and Phikon-v2 instead of Phikon). Most selected FMs are built on ViT backbones, covering multiple model scales from ViT-Small and ViT-Base to ViT-Large, ViT-Huge, and ViT-Giant, with the number of parameters ranging from 21.7M to 1.1B and embedding dimensionality ranging from 384 to 2048. These ViT-based FMs are predominantly pretrained using SSL, with DINOv2 [4] the most widely adopted self-supervised pretraining method among recent histopathology FMs. To enable comparison beyond the ViT paradigm, we additionally included FMs relying on alternative backbone architectures and SSL methods, namely, Lunit-BT [24], which uses a ResNet-50 backbone pretrained with Barlow Twins [25], and CTransPath [26], which adopts a Swin Transformer [27] backbone pretrained with MoCo-v3 [28].

The selected FMs also differ in the scale and composition of their pretraining data, ranging from tens of thousands to several million WSIs and up to billions of image tiles, and were trained on a combination of large public resources and proprietary multicenter cohorts. Pretraining magnification was most commonly 20×, while a subset of models, notably Virchow2 [6] and the Lunit models Lunit-DINO and Lunit-BT [24], were pretrained on tiles extracted at multiple magnifications. CONCH [29] differs from the other FMs by employing vision-language pretraining; in this study, only its unimodal visual encoder was used. For Virchow2, the class token representation was selected to ensure consistency with the embedding strategy adopted by the remaining models. All FMs were used in inference mode as frozen feature extractors; no fine-tuning was performed. For each selected FM, patch-level feature embeddings were computed for both the SemiCOL and BEETLE datasets following the official feature extraction guidelines provided by the original authors of each FM, including the required input resizing and image normalization, and were subsequently used for patch-level and slide-level classification tasks.

#### 2.2.3. Slide-Level Embedding (SemiCOL)

SemiCOL provides slide-level ground truth, where each WSI is associated with a single label. Accordingly, each WSI is modeled as a Multiple Instance (MI) bag composed of its extracted patch embeddings. For each selected FM, all patch-level feature vectors ${\left\{x_{i}\right\}}_{i=1}^{N}$ belonging to the same WSI are aggregated into a single slide-level representation using Simple-MI, a standard bag-to-vector transformation strategy in the MI literature [33]. Specifically, the slide embedding is computed as the arithmetic mean of all patch embeddings,

(1) $$z=\frac{1}{N}\sum_{i=1}^{N}x_{i},$$

where $x_{i}\in R^{D}$ denotes the embedding of the *i*-th patch produced by a given FM, $z\in R^{D}$ is the resulting slide-level representation, and *D* is the dimensionality of the FM embeddings. Simple-MI is adopted as a parameter-free aggregation strategy. This choice avoids introducing additional learnable pooling mechanisms and ensures that slide-level representations are obtained directly from the patch embeddings produced by the FMs, without further transformation at the aggregation stage.

#### 2.2.4. Slide-Level and Patch-Level Classification

For both datasets, classification is performed using a lightweight two-layer MLP applied to feature embeddings extracted by the FMs. The same classifier architecture is used consistently across all experiments. For SemiCOL, the MLP is applied to slide-level embeddings obtained after Simple-MI aggregation and performs binary slide-level classification. For BEETLE, the same MLP architecture is applied directly to patch-level embeddings to perform four-class tissue classification. This unified classifier design ensures a consistent evaluation protocol and avoids introducing task-specific architectural variations that could influence the comparison of FM representations.

#### 2.2.5. Evaluation Protocols

**Figure 3:**
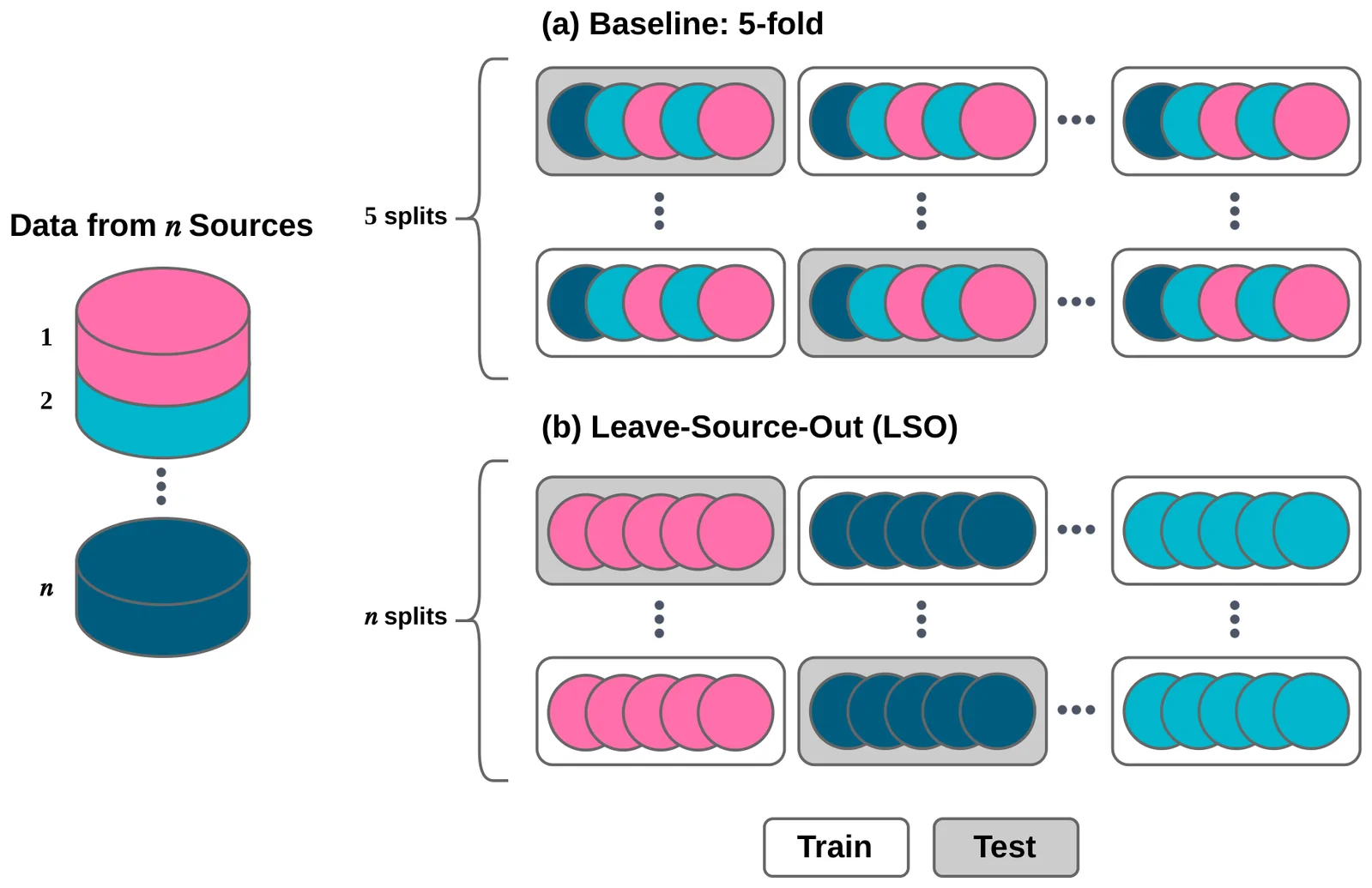
Schematic illustration of the evaluation protocols. (**a**) Baseline 5-fold CV with source-mixed random splits. (**b**) LSO-CV, where one source is held out for testing and the remaining sources are used for training.

We evaluate models using a standard 5-fold CV Baseline and leave-source-out (LSO)-CV protocols, implemented as leave-center-out (LSO-Center) and leave-scanner-out (LSO-Scanner) to assess robustness to center-induced and scanner-induced domain shifts, as shown in Figure 3.

Baseline Cross-Validation: For both SemiCOL and BEETLE, we first report Baseline performance using 5-fold stratified CV. In each fold, models are trained on four folds and evaluated on the held-out fold. For both datasets, splitting is performed at the WSI-level; for SemiCOL, slides from DSW2A and DSW2B that correspond to the same underlying specimen are always assigned to the same fold, while for BEETLE, all patches extracted from the same WSI are kept in the same fold to avoid information leakage between training and evaluation.

LSO-Center: To assess generalizability to unseen acquisition medical centers, we use an LSO-Center protocol, where each fold holds out all samples from one center for evaluation and uses the remaining centers for training. This source-level evaluation is recommended in multicenter settings, as standard random *k*-fold CV can mix sources between training and evaluation and therefore overestimate performance under center domain shift [34,35]. For SemiCOL, DSW2A and DSW2B belong to the same center (DSW2) and are grouped into a single held-out fold, resulting in four center folds (DSW1, DSW2, DSW3, DSW4). For BEETLE, we evaluate five center folds (JB, TCGA, NKI, SCH, RUMC), with each fold training on four centers and evaluating on the held-out center.

LSO-Scanner: To assess robustness to scanner-induced domain shift, we adopt an LSO-Scanner protocol, in which all samples acquired with one scanner are held out for evaluation while training is performed on data from the remaining scanners. Scanners are treated as separate domains even when originating from the same manufacturer, as different models and versions may differ in hardware characteristics, including objective lenses, illumination and focusing systems, as well as image processing and quality-control workflows, which may induce differences in sharpness, contrast, color, and texture in the digitized images. For SemiCOL, three distinct scanners are present, as shown in Table 1, resulting in three scanner folds. In each fold, models are trained on two scanners and evaluated on the held-out scanner. To prevent leakage between scanner folds, subsets DSW2A and DSW2B, which correspond to the same underlying specimens acquired with different scanners, are treated with an explicit exclusion rule: when one subset appears in the held-out fold, its paired counterpart is removed from the training set. For BEETLE, seven scanner folds are evaluated, as shown in Table 2, each corresponding to a distinct scanner model used for digitization.

## 3. Results

### 3.1. Implementation Details and Evaluation Metrics

All experiments on both datasets were trained for a maximum of 200 epochs using the Adam optimizer, with a learning rate of $1\times 10^{-5}$, a weight decay of $1\times 10^{-5}$, a dropout rate of 0.1, a batch size of 32, and a random seed of 42. All experiments were conducted using PyTorch 2.1.1 with CUDA 12.1 on an NVIDIA RTX A6000 GPU (NVIDIA Corporation, Santa Clara, CA, USA). For both datasets, no stain normalization or other domain generalization techniques were applied to the patch images, and the resulting FM embeddings were used directly for classification, without additional feature normalization. Classification was performed using a lightweight two-layer MLP with a hidden dimension of 256, chosen as a compromise to accommodate the varying dimensionality of the evaluated FM embeddings, which range from 384 to 2048 dimensions.

For the SemiCOL dataset, we consider a binary slide-level classification task optimized using the cross-entropy loss. Performance is primarily evaluated using the Area Under the ROC Curve (AUROC), with accuracy, F1-score, and sensitivity for the tumor class reported as complementary performance measures, averaged across CV folds. For the BEETLE dataset, we consider a four-class patch-level tissue classification task and train models using a weighted cross-entropy loss, with class weights computed from the training data. Evaluation for BEETLE includes overall accuracy and F1-score as primary metrics to reflect multiclass performance under class imbalance, with sensitivity and AUROC reported as complementary measures. The evaluation metrics are defined as

(2) $$\begin{array}{cc} \mathrm{Accuracy} & =\frac{1}{N}\sum_{i=1}^{N}I\left(y_{i}={\hat{y}}_{i}\right),\\ \mathrm{Sensitivity} & =\frac{\mathrm{TP}}{\mathrm{TP}+\mathrm{FN}},\\ F1\text{-}\mathrm{score} & =\frac{2\mathrm{TP}}{2\mathrm{TP}+\mathrm{FP}+\mathrm{FN}},\\ \mathrm{AUROC} & =\sum_{k=1}^{K-1}\left({\mathrm{FPR}}_{k+1}-{\mathrm{FPR}}_{k}\right)\frac{{\mathrm{TPR}}_{k+1}+{\mathrm{TPR}}_{k}}{2}. \end{array}$$

where *N* is the number of evaluated samples, $y_{i}$ and ${\hat{y}}_{i}$ are the true and predicted labels of sample *i*, respectively, and TP, FP, and FN denote the true-positive, false-positive, and false-negative counts. Furthermore, *K* is the number of operating points on the ROC curve, and ${\mathrm{TPR}}_{k}$ and ${\mathrm{FPR}}_{k}$ are the true-positive and false-positive rates at operating point *k*, respectively.

For each CV fold, accuracy is computed over all patches, F1-score and sensitivity are computed as the mean of the corresponding per-class values over the four tissue classes, and AUROC is computed as the mean of the one versus rest AUROC values over the four classes; the resulting fold-level metrics are then averaged across folds. In addition, class-wise F1-scores are reported to characterize performance for each individual tissue class. All values are reported as mean ± standard deviation. In order to quantify the effect of moving from the source-mixed Baseline protocol to the source-aware evaluation protocols, we report performance changes (Δ) for each metric relative to Baseline. For each metric *M*, changes are computed as $\Delta M_{\mathrm{Center}}=100\times \left(M_{\mathrm{LSO}\text{-}\mathrm{Center}}-M_{\mathrm{Baseline}}\right)$ and $\Delta M_{\mathrm{Scanner}}=100\times \left(M_{\mathrm{LSO}\text{-}\mathrm{Scanner}}-M_{\mathrm{Baseline}}\right)$, and reported in % units. To assess overall differences among the ten FMs, a Friedman test was applied separately to SemiCOL and BEETLE across the twelve combinations of the three evaluation protocols and four overall performance metrics, with statistical significance set at p<0.05.

### 3.2. SemiCOL: Colorectal Tumor Classification Across Centers and Scanners

**Figure 4:**
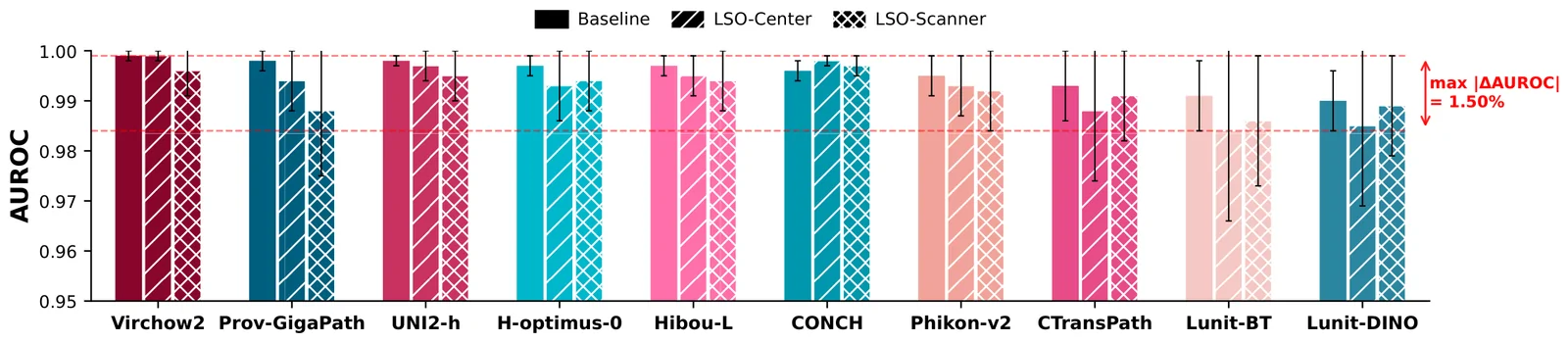
AUROC comparison across FMs on SemiCOL under the three evaluation protocols. Models are ordered by Baseline AUROC in descending order, error bars indicate the standard deviation across folds, and the dashed red lines denote the maximum absolute AUROC change across protocols (1.50%). Each FM is represented by a distinct color.

Table 5 reports slide-level classification performance on SemiCOL for each evaluated FM under the Baseline, LSO-Center, and LSO-Scanner protocols, with Δ columns quantifying differences (%) relative to Baseline when introducing acquisition-source domain shift. Figure 4 provides a compact view of the corresponding AUROC differences across protocols and the Baseline ranking of the FMs.

In the Baseline setting, all FMs achieve near-saturated performance, with AUROC ranging from 0.990 for Lunit-DINO to 0.999 for Virchow2-CLS, as shown in Figure 4. Across accuracy and F1-score, Virchow2-CLS and CONCH lead, followed by UNI2-h and Prov-GigaPath, with H-optimus-0 next; for sensitivity, the ordering is similar except that CONCH drops behind H-optimus-0. Overall, in the Baseline, differences between models remain modest, particularly in AUROC.

Under LSO-Center, performance remains close to Baseline across FMs, with $\Delta {\mathrm{AUROC}}_{\mathrm{Center}}$∈[−0.70,+0.20]%, confirming AUROC stability under center domain shift. Virchow2-CLS attains the highest AUROC, matching Baseline, and the largest AUROC decrease is observed for Lunit-BT. Accuracy and F1-score remain close to Baseline, with $\Delta {\mathrm{Acc}}_{\mathrm{Center}}$ and $\Delta F1_{\mathrm{Center}}$ both within [−0.90,+0.60]%, indicating only limited changes under center domain shift. Among the reported metrics, sensitivity shows the largest protocol effect, with $\Delta {\mathrm{SENS}}_{\mathrm{Center}}\in \left[-2.10,+1.80\right]\%$. For all evaluated FMs, the center-aware protocol induces below 1% changes in AUROC, accuracy, and F1-score, while the larger variability concentrates in sensitivity. CONCH is the only model that improves across all four metrics under LSO-Center, indicating a small net gain relative to Baseline.

Under LSO-Scanner, performance remains close to Baseline across FMs, with gaps that are slightly wider than under LSO-Center for some models. Overall, $\Delta {\mathrm{AUROC}}_{\mathrm{Scanner}}\in \left[-1.00,+0.10\right]\%$, with CONCH ranking first; the largest AUROC decrease is observed for Prov-GigaPath (−1.00%). Accuracy and F1-score are jointly led by CONCH and Virchow2-CLS at 0.980 for both metrics, matching their LSO-Center values. These differences lie within [−1.80,+0.30]% for both $\Delta {\mathrm{Acc}}_{\mathrm{Scanner}}$ and $\Delta F1_{\mathrm{Scanner}}$; the largest decreases in both metrics occur for Prov-GigaPath (−1.80%). Compared with the other metrics, sensitivity shows the clearest scanner shift effect, with $\Delta {\mathrm{SENS}}_{\mathrm{Scanner}}\in \left[-3.60,+1.20\right]\%$; the largest decrease occurs for Prov-GigaPath (−3.60%).

Across the source-aware protocols, changes in AUROC, accuracy, and F1-score remain within 2% across FMs, with slightly wider ranges under LSO-Scanner than under LSO-Center; sensitivity shows the largest absolute deviations (up to 3.6%). Additionally, across the three evaluation protocols and four overall performance metrics, the Friedman test indicated a statistically significant overall difference among the ten FMs ($\chi^{2}\left(9\right)=85.26$, p<0.001), with Virchow2-CLS achieving the highest overall ranking. Overall, performance on SemiCOL shows limited separation between models under source-held-out evaluation, as most protocol-induced changes are small, making this binary task a weak stress test of robustness to acquisition-source domain shift.

### 3.3. BEETLE: Breast Tissue Classification Across Acquisition Domains

**Figure 5:**
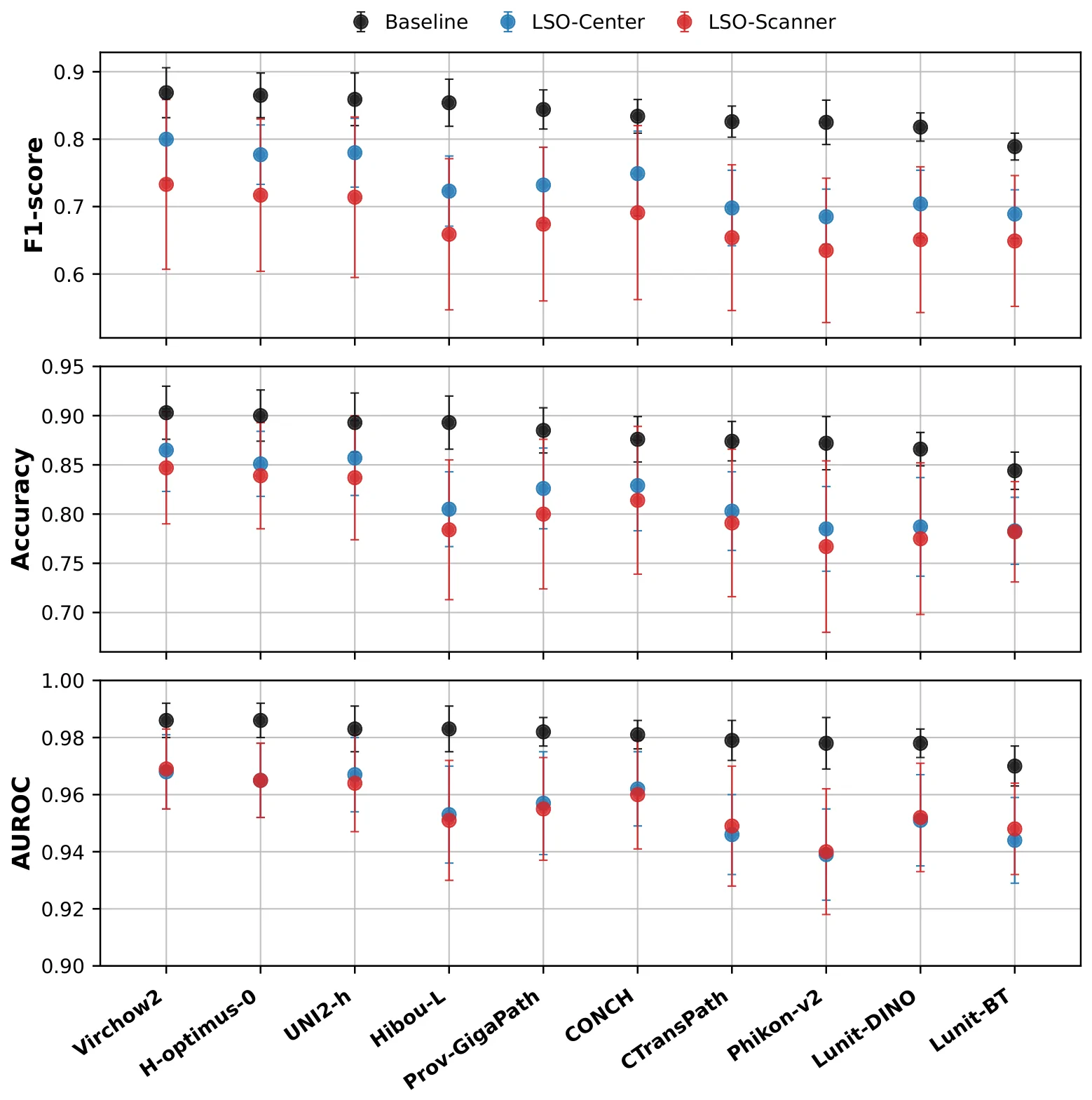
BEETLE overall performance comparison across FMs under the three evaluation protocols, reported as mean ± standard deviation for F1-score, accuracy, and AUROC. Models are ordered by Baseline F1-score in descending order.

Table 6 summarizes patch-level classification performance on the BEETLE dataset for each evaluated FM, including overall accuracy, F1-score, AUROC, sensitivity, and class-wise F1-scores for the four tissue classes, under the Baseline 5-fold CV protocol and the source-aware LSO-Center and LSO-Scanner settings. Table 7 reports the corresponding performance gaps as differences (%) for these metrics; $\Delta_{\mathrm{Center}}$ and $\Delta_{\mathrm{Scanner}}$ are computed relative to Baseline under LSO-Center and LSO-Scanner, respectively. Figure 5 complements these tables by visualizing the three overall metrics for all models under the evaluation protocols, making their comparative behavior easier to read.

#### 3.3.1. Overall Generalization Gaps Under Domain Shift

Under the Baseline setting, performance ranges are as follows: Accuracy∈[0.844,0.903], F1-score∈[0.789,0.869], and Sensitivity∈[0.838,0.895]. Notably, AUROC remains within a narrow range of [0.970,0.986]. Across these metrics, Virchow2-CLS ranks first, followed by H-optimus-0, with UNI2-h and Hibou-L next, while Lunit-BT is the lowest; this ranking is also reflected in Figure 5, where models are ordered by Baseline F1-score in descending order. Given this limited variation in AUROC, accuracy and F1-score better capture performance differences between the evaluated FMs for this multiclass task.

Under LSO-Center, all FMs show lower performance than Baseline across the four overall metrics (Figure 5). In particular, the performance gaps range between $\Delta {\mathrm{Acc}}_{\mathrm{Center}}\in \left[-8.78,-3.64\right]\%$, $\Delta F1_{\mathrm{Center}}\in \left[-14.02,-6.92\right]\%$, and $\Delta {\mathrm{SENS}}_{\mathrm{Center}}\in \left[-12.06,-5.13\right]\%$. In contrast, AUROC is less affected, with $\Delta {\mathrm{AUROC}}_{\mathrm{Center}}\in \left[-3.81,-1.64\right]\%$ across models. UNI2-h and Virchow2-CLS are the most robust to the center domain shift, with UNI2-h leading in accuracy and AUROC stability and Virchow2-CLS leading in F1-score and sensitivity stability. Phikon-v2 shows the largest losses in F1-score, sensitivity, and AUROC, while Hibou-L has the largest $\Delta {\mathrm{Acc}}_{\mathrm{Center}}$.

Under LSO-Scanner, performance is lower than Baseline, and the gaps are typically larger than under LSO-Center (Figure 5). Across models, the performance gaps range between $\Delta {\mathrm{Acc}}_{\mathrm{Scanner}}\in \left[-10.86,-5.59\right]\%$, $\Delta F1_{\mathrm{Scanner}}\in \left[-19.53,-13.59\right]\%$, and $\Delta {\mathrm{SENS}}_{\mathrm{Scanner}}\in \left[-14.41,-8.53\right]\%$. In contrast, AUROC is less affected, with its gap relative to Baseline falling within [−3.72,−1.74]% under LSO-Scanner. Virchow2-CLS is among the most robust to scanner domain shift, achieving the smallest $\Delta F1_{\mathrm{Scanner}}$ and $\Delta {\mathrm{AUROC}}_{\mathrm{Scanner}}$, and sharing the smallest $\Delta {\mathrm{Acc}}_{\mathrm{Scanner}}$ with UNI2-h. In contrast, Hibou-L has the largest $\Delta {\mathrm{Acc}}_{\mathrm{Scanner}}$ and $\Delta F1_{\mathrm{Scanner}}$, while Phikon-v2 has the largest $\Delta {\mathrm{SENS}}_{\mathrm{Scanner}}$ and $\Delta {\mathrm{AUROC}}_{\mathrm{Scanner}}$, together indicating the weakest generalization under scanner hold-out across the overall metrics. Across the three evaluation protocols and four overall performance metrics, the Friedman test indicated a statistically significant overall difference among the ten FMs ($\chi^{2}\left(9\right)=97.16$, p<0.001), with Virchow2-CLS achieving the best overall rank.

Compared with LSO-Center, scanner domain shift leads to larger degradations in accuracy, F1-score, and sensitivity, while AUROC remains comparatively stable, showing that conventional source-mixed CV can considerably overestimate real-world performance. Additionally, Figure 5 highlights the increased uncertainty under the source-aware protocols. The error bars (standard deviation) widen under the source-aware protocols across the three plotted metrics, most clearly under LSO-Scanner, indicating higher split-to-split variability as the held-out center or scanner changes across splits.

**Figure 6:**
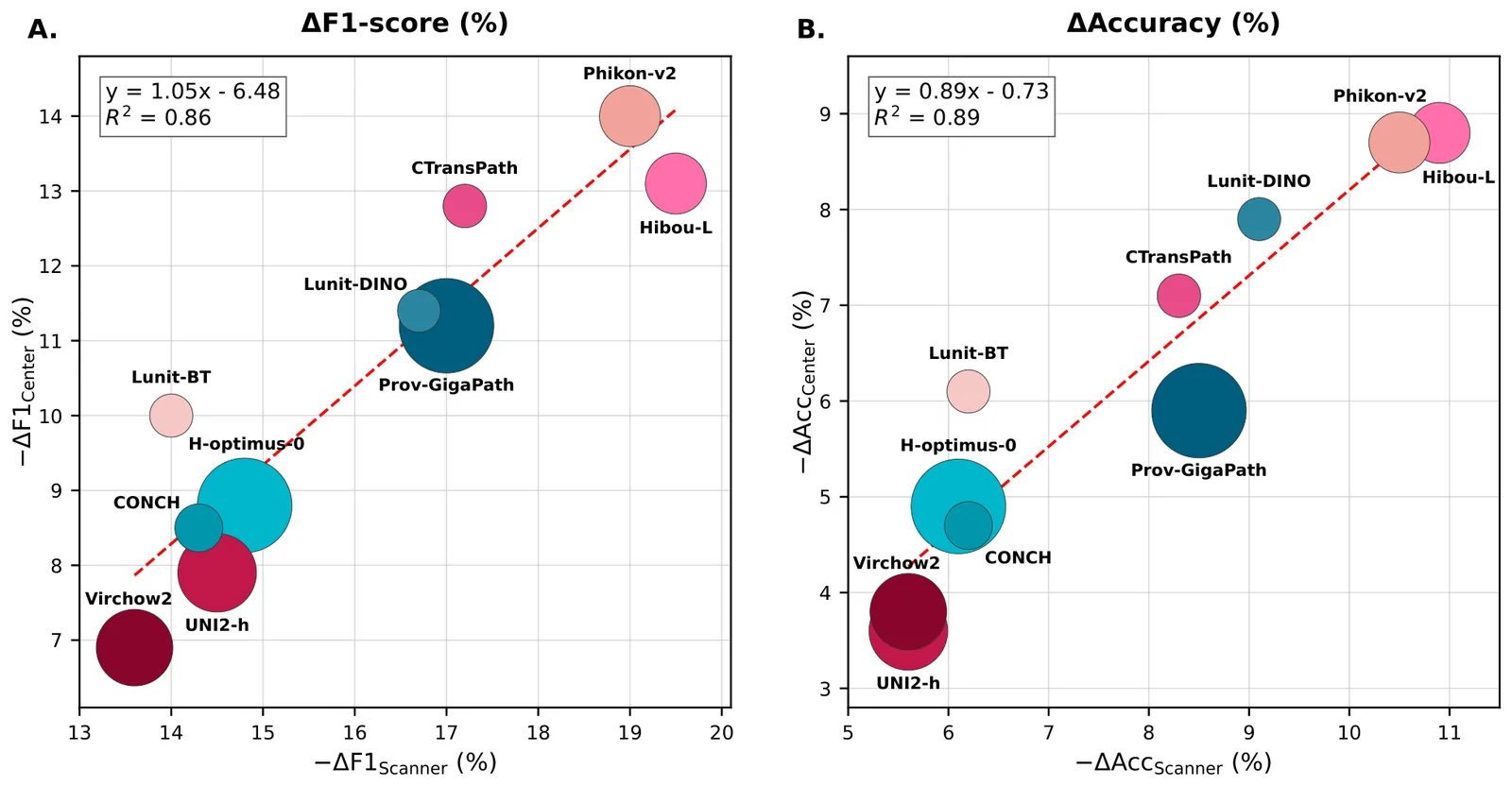
BEETLE joint robustness analysis across FMs. Each circle denotes the performance loss (%) relative to the Baseline for (**A**) F1-score and (**B**) accuracy, reported as $-\Delta_{\mathrm{Scanner}}$ and $-\Delta_{\mathrm{Center}}$. Circle area is proportional to the number of model parameters (millions), and the red line indicates the linear trend.

Figure 6 provides a joint robustness map by placing each FM according to its paired gaps, reported as $-\Delta_{\mathrm{Scanner}}$ (x-axis) and $-\Delta_{\mathrm{Center}}$ (y-axis), which represent the performance degradations (%) under LSO-Scanner and LSO-Center relative to the Baseline setting for (A) F1-score and (B) accuracy. We plot the negated gaps so that larger degradations move rightwards and upwards in the figure, which makes the robustness ordering easier to interpret. This visualization facilitates comparison of robustness trends across models; Virchow2-CLS lies closest to the origin (0,0) overall, UNI2-h is comparably close in accuracy stability, and Hibou-L and Phikon-v2 concentrate farther from the origin along both axes, indicating the strongest instability across both domains for F1-score and accuracy. Among the two Lunit variants, the gaps relative to the Baseline protocol are smaller for Lunit-BT than for Lunit-DINO in F1-score and accuracy in both LSO settings, suggesting better robustness for Lunit-BT than for Lunit-DINO. Based on the x- and y-axis gaps, the scanner domain appears more challenging than the center domain, as models deviate further from zero along $-\Delta_{\mathrm{Scanner}}$ than along $-\Delta_{\mathrm{Center}}$. Finally, circle area encodes model size (parameter count), showing that robustness is not correlated with scale; larger models are not systematically closer to zero, and smaller models can be among the most robust across both evaluation domains, for example, Prov-GigaPath is larger in terms of parameter count than Virchow2-CLS yet shows lower robustness in our experiments.

#### 3.3.2. Class-Wise Generalization Patterns

Class-wise F1-scores in Table 6, together with the gap table in Table 7, show that, across models, the two epithelial classes, Non-Invasive and Invasive, experience larger decreases than Other and Necrosis when moving from the Baseline protocol to the source-aware settings, which explains much of the overall F1-score drop.

Across models, Other is the most stable class, with $\Delta F1_{\mathrm{Center}}\in \left[-5.18,-2.63\right]\%$ and $\Delta F1_{\mathrm{Scanner}}\in \left[-6.00,-3.05\right]\%$ relative to the Baseline protocol. This behavior is expected, as Other acts as a residual class that aggregates heterogeneous tissue patterns outside the epithelial and necrotic classes; moreover, Other is the most represented class in BEETLE, as shown in Table 3, with 51.3k patches out of 97.8k, providing models with considerably more training examples for this class and making its class-wise performance estimates more reliable across evaluation protocols.

Non-Invasive is the main driver of the overall F1-score decrease under the source-aware protocols, with $\Delta F1_{\mathrm{Center}}\in \left[-36.91,-18.59\right]\%$ and $\Delta F1_{\mathrm{Scanner}}\in \left[-37.83,-23.21\right]\%$. Virchow2-CLS remains the strongest model for this class across all protocols, while Phikon-v2 shows the largest degradation under both source-aware settings and becomes the weakest under LSO-Scanner. For this class, Lunit-BT has the lowest F1-score in the Baseline and LSO-Center protocols, yet it shows smaller $\Delta F1_{\mathrm{Center}}$ and $\Delta F1_{\mathrm{Scanner}}$ than CTransPath, Hibou-L, Lunit-DINO, and Phikon-v2.

Compared with Non-Invasive, Invasive shows smaller F1-score degradations under the source-aware protocols. Across models, the degradation falls within $\Delta F1_{\mathrm{Center}}\in \left[-15.35,-4.36\right]\%$ and $\Delta F1_{\mathrm{Scanner}}\in \left[-27.90,-17.67\right]\%$ under center-induced and scanner-induced domain shift, respectively. Under LSO-Center, UNI2-h has the lowest $\Delta F1_{\mathrm{Center}}$ for this class, followed by Virchow2-CLS, whereas the largest drops are observed for Hibou-L, followed by Phikon-v2 and CTransPath. Under LSO-Scanner, all models degrade further; Lunit-BT has the lowest $\Delta F1_{\mathrm{Scanner}}$ for Invasive, followed by UNI2-h and Virchow2-CLS, while the largest degradations are observed for Hibou-L and Phikon-v2.

Necrosis shows smaller changes than the epithelial classes, with $\Delta F1_{\mathrm{Center}}\in \left[-2.78,+4.03\right]\%$ and $\Delta F1_{\mathrm{Scanner}}\in \left[-11.10,-2.79\right]\%$. Under LSO-Center, several models achieve higher Necrosis F1-scores than in the Baseline protocol, led by Lunit-DINO, followed by Lunit-BT, CONCH, and Hibou-L, whereas the largest drops are observed for Prov-GigaPath and UNI2-h. In contrast, $\Delta F1_{\mathrm{Scanner}}$ is negative for all models; Lunit-BT shows the smallest gap, followed by Lunit-DINO and CONCH, while the strongest degradations are observed for UNI2-h. As shown in Table 3, Necrosis is the least represented class, with 4.2k patches out of 97.8k; nevertheless, its F1-score gaps under both source-held-out protocols relative to the Baseline protocol remain clearly smaller than those of Non-Invasive and Invasive, indicating that center and scanner domain shifts affect epithelial discrimination more strongly than necrotic tissue recognition.

**Figure 7:**
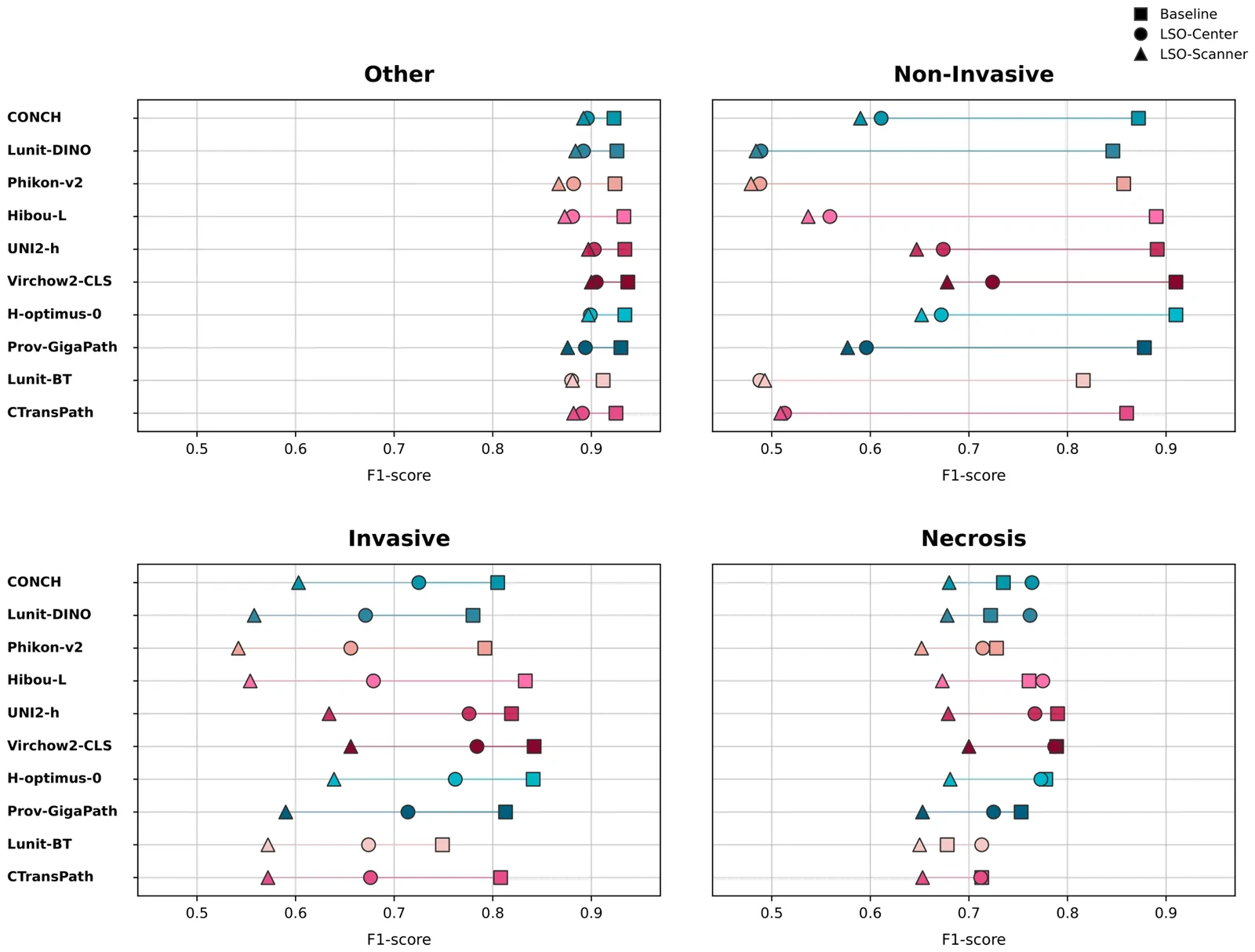
BEETLE class-wise F1-score comparison across FMs under the Baseline, LSO-Center, and LSO-Scanner protocols. Each FM is represented by a distinct color.

Figure 7 summarizes the class-wise robustness patterns by reporting, for each model and tissue class, the Baseline F1-score (squares) together with the corresponding LSO-Center (circles) and LSO-Scanner (triangles) results on the same horizontal axis. The within-row horizontal separation between markers directly reflects the degradation under acquisition-source domain shift, and shows that the effect is strongly class-dependent. For Other, the LSO-Center and LSO-Scanner markers are nearly overlapping for every model; the additional degradation from center to scanner is small, with $\left(\Delta F1_{\mathrm{Scanner}}-\Delta F1_{\mathrm{Center}}\right)\in \left[-1.85,0.14\right]\%$ (mean =−0.745%). Both source-aware markers are shifted left relative to the Baseline squares, indicating a performance drop; however, their close spacing shows that scanner shifts do not introduce much more instability than center shifts for this class. In line with Table 7, this visualization confirms that the largest Baseline-to-source-aware separations occur for Non-Invasive, where the circle–triangle spacing is often limited (mean $(\Delta F1_{\mathrm{Scanner}}-\Delta F1_{\mathrm{Center}})=-1.68\%$); compared with Non-Invasive, Invasive shows smaller Baseline-to-source-aware drops overall but a larger center-to-scanner separation, with $\left(\Delta F1_{\mathrm{Scanner}}-\Delta F1_{\mathrm{Center}}\right)\in \left[-14.22,-10.20\right]\%$. Necrosis remains relatively stable under center domain shift; several models show near-overlap between the Baseline squares and the circles, and the change can even be slightly positive (up to +4.03%), with a near-zero mean change across models relative to Baseline (mean $\Delta F1_{\mathrm{Center}}=+0.45\%$). In contrast, scanner domain shift introduces an additional degradation, with $\left(\Delta F1_{\mathrm{Scanner}}-\Delta F1_{\mathrm{Center}}\right)\in \left[-10.21,-5.88\right]\%$ (mean =−7.94%), and an average drop of −7.49% relative to Baseline. Overall, the class-wise patterns indicate that domain shifts mainly challenge epithelial discrimination.

**Figure 8:**
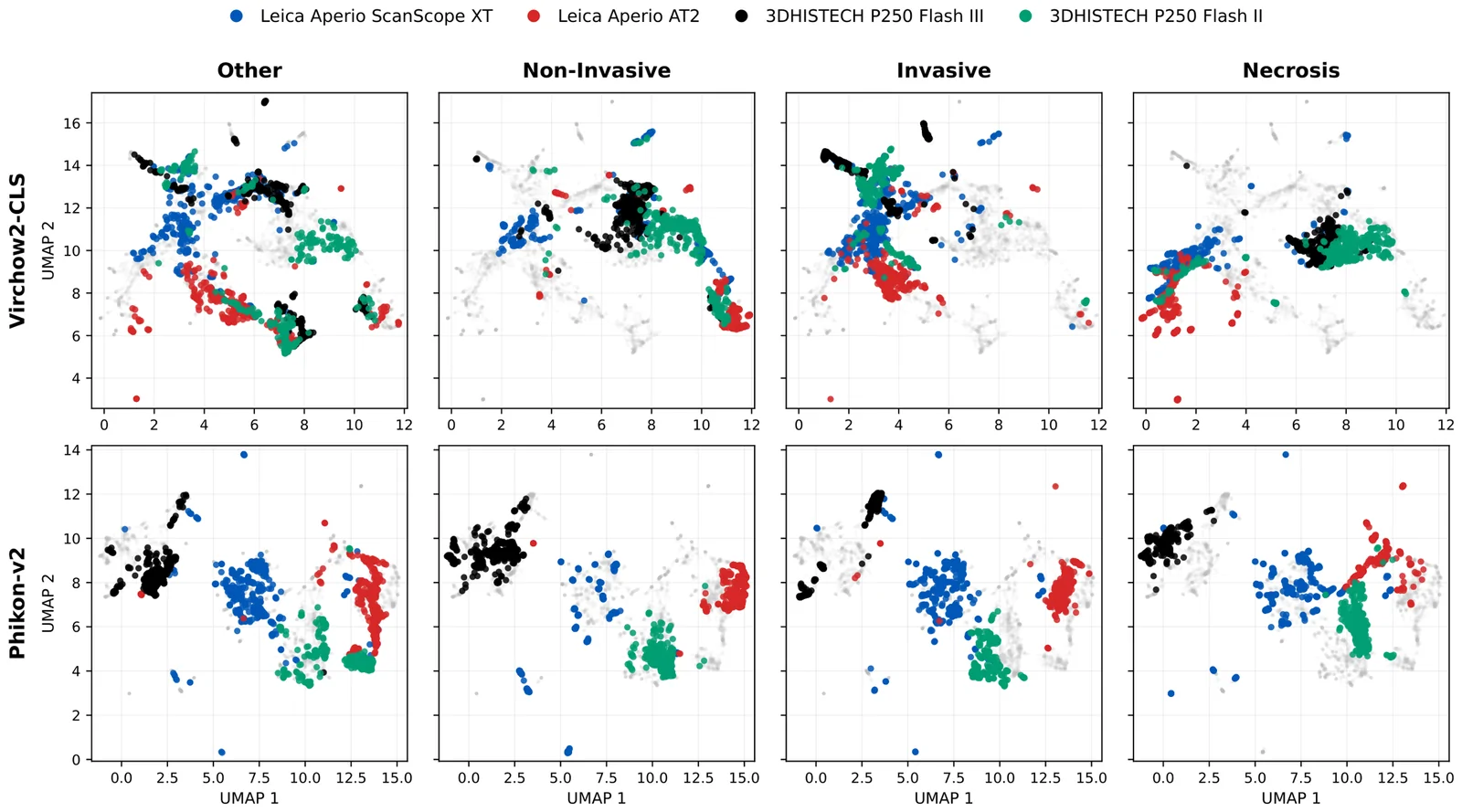
UMAP visualization of Virchow2-CLS and Phikon-v2 BEETLE patch embeddings using a balanced subset across four tissue classes and four scanners. Patches are colored by scanner within each tissue class.

To visually assess scanner bias in the feature space, Figure 8 shows 2D UMAP [36] projections of the Virchow2-CLS and Phikon-v2 patch embeddings. These FMs were selected as representative models at opposite ends of performance under the source-aware protocols. Because the patch distribution is imbalanced across scanners and tissue classes (Table 3), we retained the four scanners containing sufficient samples in all four classes (Leica Aperio ScanScope XT, Leica Aperio AT2, 3DHISTECH P250 Flash III, and 3DHISTECH P250 Flash II) and selected a balanced sample of 418 patches per class and scanner, corresponding to the minimum class-specific patch count across these four scanners. The same patches were used for both FMs. The projections were computed separately for each FM using the cosine metric. Phikon-v2 shows clearer clustering and separation of patch embeddings according to scanner within the feature space, whereas the corresponding Virchow2-CLS embeddings show greater inter-scanner mixing, consistent with their relative robustness under LSO-Scanner.

**Figure 9:**
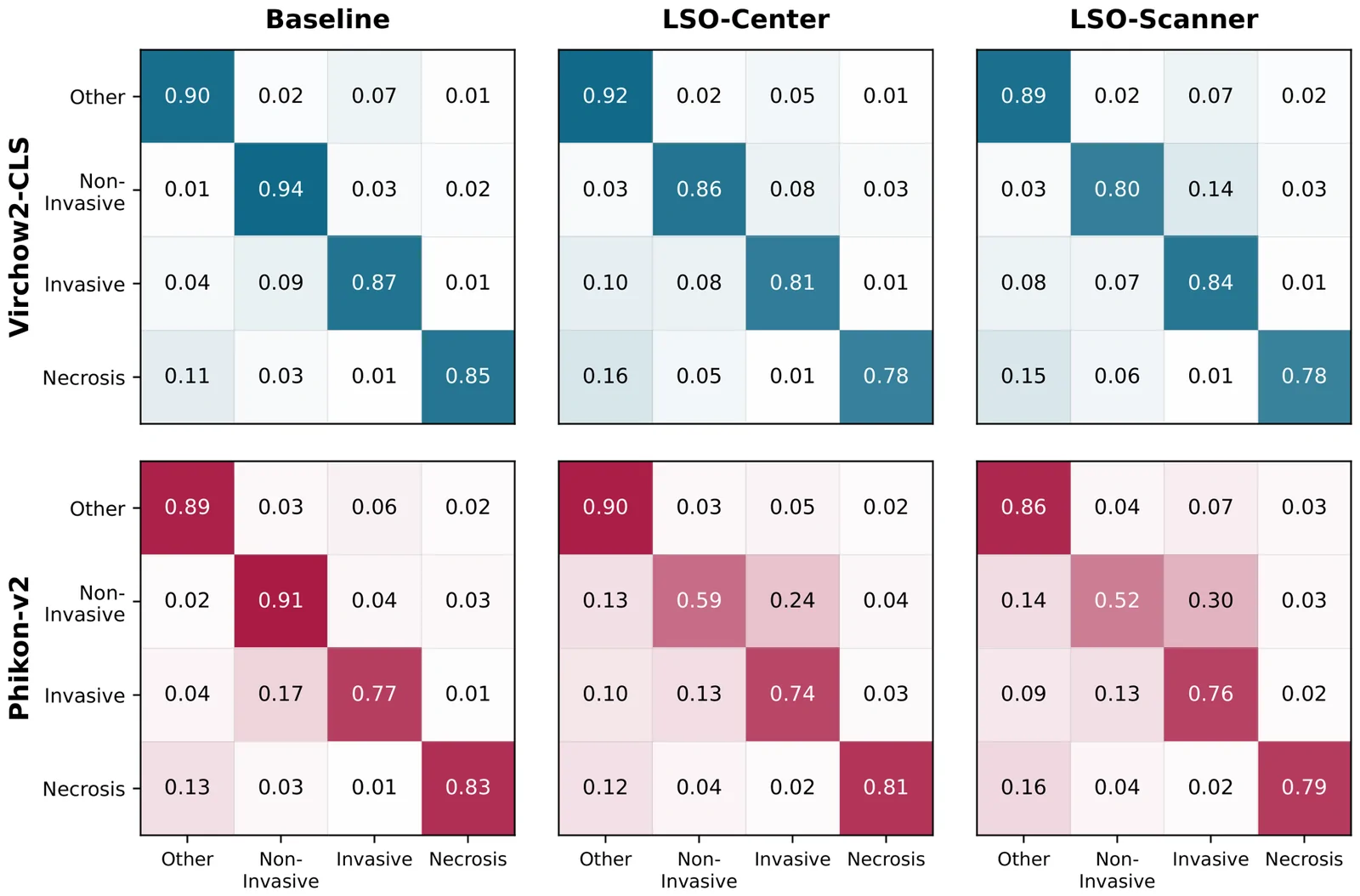
Confusion matrices for *Virchow2-CLS* and *Phikon-v2* patch classification on BEETLE under the three evaluation protocols.

The confusion matrices in Figure 9 further explain these class-wise trends for the same two FMs. Virchow2-CLS achieves the highest overall F1-score and Phikon-v2 the lowest under LSO-Center and LSO-Scanner; this choice provides an interpretable view of typical error modes without reporting confusion matrices for all models. For both models, the main error pattern under source-aware protocols is an increase in confusion where Non-Invasive is predicted as Invasive. For Virchow2-CLS, the proportion of true Non-Invasive patches predicted as Invasive increases from 0.028 in the Baseline setting to 0.082 under LSO-Center and 0.141 under LSO-Scanner. The same trend is stronger for Phikon-v2, where the proportion of true Non-Invasive patches predicted as Invasive increases from 0.039 in the Baseline setting to 0.244 under LSO-Center and 0.304 under LSO-Scanner. In addition, Phikon-v2 shows a marked increase in epithelial patches being assigned to Other under the source-aware protocols, indicating reduced separation between epithelial tissue and non-epithelial patterns grouped under Other. These changes align with the large decrease in Non-Invasive F1-score reported for Phikon-v2 in Table 6. Overall, the confusion matrices confirm that source-aware evaluation mainly affects fine-grained epithelial discrimination, while Other remains relatively stable and Necrosis is less affected than the epithelial classes, with most errors corresponding to Necrosis being predicted as Other.

## 4. Discussion

This benchmark evaluated domain generalization of histopathology FMs under multi-source acquisition variability on two tasks, binary slide-level tumor status prediction on SemiCOL and four-class patch-level tissue prediction on BEETLE. Each task was assessed under a Baseline 5-fold CV protocol and source-aware protocols, implemented as LSO-Center and LSO-Scanner. Across the two datasets, the usefulness of domain-aware evaluation depends on whether the task is challenging enough to expose cross-domain generalization failures. When performance is near-saturated on a dataset, as in SemiCOL, ranking the evaluated FMs by robustness becomes hard to interpret. In contrast, on the more demanding BEETLE setting, center-held-out and scanner-held-out evaluation reveals clear generalization gaps that are hidden by source-mixed CV. This contrast should still be read with care, as the two tasks differ in several respects beyond difficulty (e.g., organ, label granularity, and supervision).

On SemiCOL, the Baseline results show near-saturated performance for binary slide-level Tumor vs. Benign classification across all evaluated histopathology FMs, with AUROC close to 1, alongside high accuracy and F1-score, with only slight variation across models. The source-aware LSO-Center and LSO-Scanner protocols introduce only small deviations from Baseline, with sensitivity showing the largest relative variation. SemiCOL therefore indicates that slide-level tumor status prediction remains reliable for all evaluated FMs under center and scanner domain shift, but it is less informative for ranking generalization under acquisition-source domain shift, likely because the task is easy for the in-domain histopathology representations of these FMs. Overall on SemiCOL, Virchow2-CLS achieves the strongest performance, with UNI2-h and Prov-GigaPath close behind in the Baseline AUROC, while CONCH remains among the top models under the source-aware protocols.

On BEETLE, the four-class patch-level tissue classification task reveals clear generalization gaps in overall metrics that are hidden by source-mixed Baseline CV. Relative to Baseline, LSO-Center reduces overall accuracy and F1-score for every evaluated FM, and degradations are even larger under LSO-Scanner. These results show that source-mixed Baseline CV can overestimate FM domain generalizability, and that scanner-induced domain shift is more challenging than center-induced domain shift in this patch-level tissue classification task. Moreover, the higher standard deviation under the source-aware protocols reflects that held-out sources differ in sample size and acquisition setting (Table 2), with the Leica Aperio GT 450 DX scanner also lacking Invasive annotations; therefore, the LSO folds vary in difficulty, an effect that random source-mixed splits can hide. Overall on BEETLE, Virchow2-CLS and UNI2-h emerge as the strongest and most robust FMs under acquisition-source domain shift (Figure 6). In contrast, Hibou-L and Phikon-v2 show the largest performance losses under domain shift. The BEETLE TCGA subset includes one diagnostic WSI from each of 164 TCGA-BRCA patients, representing approximately 15.4% of the full TCGA-BRCA cohort, which comprises 1,062 cases. Because each patient may have one or more diagnostic WSIs and the specific TCGA cases and slides used to pretrain each FM are not publicly reported, the exact overlap cannot be determined. If some of these slides were included during pretraining, the reported BEETLE performance across the evaluation protocols could be inflated, making the domain-generalization results appear more optimistic than they would on fully unseen data.

Class-wise results and confusion matrices (Figure 7 and Figure 9) indicate that robustness degradation when evaluating generalization to unseen acquisition sources is strongly class-dependent and concentrates in epithelial discrimination. The Other class shows the most stable performance under acquisition-source domain shift, which is expected given its role as a residual class and the fact that it is the most represented class in BEETLE. In contrast, the largest degradations occur for Non-Invasive, which is the main driver of the overall F1-score decrease, and under source-aware evaluation a growing proportion of Non-Invasive patches are predicted as Invasive, with stronger effects under scanner domain shift. Necrosis is less affected than both epithelial classes despite being the least represented class, with most errors corresponding to Necrosis being predicted as Other. Overall, the dominant confusion between Non-Invasive and Invasive under acquisition-source domain shift is clinically important, as it can affect breast cancer staging and treatment planning. This suggests that acquisition domain shift primarily disrupts the fine-grained morphological features needed to separate these visually similar epithelial classes, while broader tissue categorization (e.g., Other and Necrosis) remains comparatively stable. Distinguishing Non-Invasive from Invasive epithelium depends on recognizing whether epithelial structures remain confined or extend into the surrounding stroma, and scanner-related differences, including color reproduction, contrast, sharpness, and resolution, may affect the visibility of these subtle architectural cues. These patterns indicate that robustness under acquisition shift is governed less by class frequency than by the morphological distinctness of each class.

Across FMs, robustness under source-aware evaluation on BEETLE shows no clear correlation with parameter count (Figure 6). Larger models do not necessarily show higher robustness to domain shift, and smaller models can remain among the most robust. More generally, robustness to domain shift cannot be explained by a single pretraining factor, since model scale, backbone family, SSL objective, pretraining data scale and provenance, and pretraining magnification vary across models (Table 4). The Lunit variants provide a concrete example, as Lunit-BT (ResNet-50, Barlow Twins, 2048-dimensional) shows higher robustness to domain shift than Lunit-DINO (ViT-S, DINOv1, 384-dimensional) under source-aware evaluation, even though both are pretrained on the same data sources, highlighting that the backbone family and SSL objective are correlated with FMs robustness to center and scanner domain shift. In addition, most evaluated FMs are pretrained on proprietary in-house datasets, and the majority rely on ViT backbones and DINOv2-based SSL objectives, which limits direct attribution of robustness differences to data composition, backbone family, or SSL objective within this benchmark. Finally, Virchow2-CLS is the only model pretrained at four magnifications (5×, 10×, 20×, 40×) and achieves high overall performance and robustness across both tasks and tissue types evaluated in this benchmark, suggesting a possible association between multi-scale pretraining and both performance and robustness to acquisition domain shift, although this benchmark does not isolate magnification as a causal factor.

In this benchmark, we did not apply any domain-shift mitigation methods. Existing approaches include image-level methods, such as stain normalization (e.g., Reinhard or Macenko) and color or stain augmentation [17,18], as well as task-specific adaptation of FM representations. The latter can be implemented through additional training objectives, as in FLEX [20] and the contrastive ScanGen loss [21]. Prior work indicates that such methods may reduce the center- and scanner-related gaps observed here, although the reported reductions range from limited to substantial depending on the task and method [17,18,19].

Taken together, these findings highlight the need to assess FM domain generalization across acquisition centers and scanners, as source-mixed CV can mask performance gaps on unseen acquisition sources. In this benchmark, scanner-related effects on FM performance are generally more pronounced than center-related effects. However, this interpretation should take into account that scanner and center effects are not fully independent in multi-source datasets, because images acquired with a given scanner also reflect the tissue processing, staining, and acquisition workflows of the center in which it is used; therefore, the observed scanner-related effects may partly reflect institutional differences. In addition, acquisition-related generalization gaps were more visible on the more demanding BEETLE setting, suggesting that harder tasks provide clearer tests of robustness to domain shift.

## 5. Limitations

This benchmark is scoped to colorectal slide-level tumor classification and breast cancer patch-level tissue classification on SemiCOL and BEETLE. Consequently, the robustness trends reported here may differ for other organs or other CPath tasks (e.g., survival analysis). Also, we used 512×512 patches for both tasks, which may influence the reported performance, and results may differ under alternative patch sizes. In addition, both datasets are provided at 20× magnification, which aligns with typical pretraining settings for most evaluated FMs; thus, the reported trends may differ in multi-scale settings or at other magnifications. Moreover, performance on SemiCOL is near-saturated for most evaluated FMs across protocols, which limits its ability to separate models in terms of robustness under source-held-out evaluation. Our comparison follows an ablation-style setting where the same fixed MLP classifier head is used for all FMs to keep the benchmark controlled and comparable. Because FMs produce embeddings with different dimensionalities, a fixed head may not be equally well matched to every representation, which can affect the reported performance. Accordingly, the findings should be interpreted within the controlled benchmark setting adopted here, in which pretrained FMs are kept frozen, while alternative benchmark configurations, such as fine-tuning or learned aggregation, were not evaluated in this study and may lead to different performance patterns across FMs.

## 6. Conclusions

This benchmark evaluates domain generalization of ten state-of-the-art histopathology FMs under center- and scanner-related acquisition variability on SemiCOL and BEETLE. It shows that robustness claims based on source-mixed Baseline CV can be misleading in multicenter, multi-scanner histopathology data, and can overestimate cross-source generalizability. For binary slide-level tumor status classification, as in SemiCOL, the evaluated histopathology FMs maintain high performance when tested under domain shift across acquisition sources, with Virchow2 achieving the strongest overall performance, followed by CONCH and UNI2-h. In contrast, for patch-level multi-class tissue classification, as in BEETLE, the same protocols reveal a clear performance loss and expose generalizability gaps that are hidden in source-mixed CV. Virchow2 and UNI2-h remain the most robust under center and scanner domain shifts, whereas Hibou-L and Phikon-v2 show the largest performance losses. Consequently, FM selection is critical in multi-source histopathology cohorts, and this benchmark provides an evidence-based ranking of FM domain generalization, identifying models that remain stable under center and scanner domain shifts. Future work will extend the analysis to additional organs and tasks, include multi-magnification settings, and evaluate whether domain generalization techniques, such as stain normalization and stain augmentation, reduce the FM acquisition-source gaps observed in this benchmark.

## Figures and Tables

**Table 1:** Distribution of the SemiCOL weakly labeled WSIs across centers and scanners (T: Tumor, B: Benign).

Center ID	Center	WSIs (T/B)	Scanner
DSW1	University Hospital Cologne	100 (50/50)	Hamamatsu NZ S360
DSW2A	Hospital Wiener Neustadt	100 (50/50)	Hamamatsu NZ S360
DSW2B	Hospital Wiener Neustadt	100 (50/50)	Leica Aperio GT 450
DSW3	University Hospital LMU Munich	100 (50/50)	Leica Aperio GT 450
DSW4	University Hospital Bern	99 (50/49)	3DHISTECH P1000
Total WSIs		499 (250/249)	

**Table 2:** Distribution of BEETLE WSIs across centers and scanners.

Center ID	Center	WSIs	Scanner
JB	Jules Bordet Institute	18	Hamamatsu NZ 2.0 RS
TCGA	The Cancer Genome Atlas	164	Leica Aperio ScanScope XT
NKI	Netherlands Cancer Institute	113	Leica Aperio AT2
SCH	Santa Chiara Hospital	35	3DHISTECH P250 Flash III
SCH	Santa Chiara Hospital	20	Leica Aperio GT 450 DX
RUMC	Radboud University Medical Center	170	3DHISTECH P250 Flash II
RUMC	Radboud University Medical Center	67	3DHISTECH P1000
Total WSIs		587	

**Table 3:** Patch-level distribution of the BEETLE dataset across centers, scanners, and tissue classes.

Center ID	Scanner	Other	Non- Invasive	Invasive	Necrosis	Total
JB	Hamamatsu NZ 2.0 RS	343	39	132	22	536
TCGA	Leica Aperio ScanScope XT	5845	444	4426	456	11,171
NKI	Leica Aperio AT2	2383	418	2094	612	5507
SCH	3DHISTECH P250 Flash III	10,246	3186	678	633	14,743
SCH	Leica Aperio GT 450 DX	2015	492	0	93	2600
RUMC	3DHISTECH P250 Flash II	23,665	17,955	9950	2392	53,962
RUMC	3DHISTECH P1000	6782	276	2183	36	9277
Total patches		51,279	22,810	19,463	4244	97,796

**Table 4:** Overview of the ten histopathology FMs selected for this benchmark and their pretraining settings. Information not reported is marked by (–).

Model	SSL	Backbone	Params	WSIs (Tiles)	Pretraining Data	Pretraining Magnification	Embedding Dimension
CONCH [29]	iBOT	ViT-B	90M	21.4k (16M)	Proprietary	20×	512
Lunit-DINO [24]	DINOv1	ViT-S	21.7M	36.7k (32.6M)	TCGA, Proprietary	20×, 40×	384
Phikon-v2 [30]	DINOv2	ViT-L	307M	58.4k (456M)	PANCAN-XL	20×	1024
Hibou-L [31]	DINOv2	ViT-L	307M	1.14M (1.2B)	Proprietary	20×	1024
UNI2-h [32]	DINOv2	ViT-H	681M	350k (200M)	Proprietary	20×	1536
Virchow2-CLS [6]	DINOv2	ViT-H	632M	3.1M (2B)	Proprietary	5×, 10×, 20×, 40×	1280
H-optimus-0 [9]	DINOv2/iBOT	ViT-G	1.1B	500k (–)	Proprietary	20×	1536
Prov-GigaPath [8]	DINOv2	ViT-G	1.1B	170k (1.3B)	Proprietary	20×	1536
Lunit-BT [24]	Barlow Twins	ResNet-50	23.6M	36.7k (32.6M)	TCGA, Proprietary	20×, 40×	2048
CTransPath [26]	MoCo-v3	Swin-T	28M	32.2k (16M)	TCGA, PAIP	20×	768

**Table 5:** SemiCOL slide-level classification performance under the Baseline 5-fold CV protocol and the source-aware protocols LSO-Center and LSO-Scanner. Values are reported as mean ± standard deviation, and Δ columns denote differences (%) relative to the Baseline.

Model	Setting	AUROC		Accuracy		F1-Score		Sensitivity	
		Mean ± SD	Δ (%)	Mean ± SD	Δ (%)	Mean ± SD	Δ (%)	Mean ± SD	Δ (%)
CONCH	Baseline	0.996±0.002		0.978±0.013		0.977±0.014		0.963±0.028	
	LSO-Center	0.998±0.001	+0.20%	0.980±0.012	+0.20%	0.980±0.013	+0.30%	0.970±0.030	+0.70%
	LSO-Scanner	0.997±0.002	+0.10%	0.980±0.004	+0.20%	0.980±0.004	+0.30%	0.967±0.017	+0.40%
Lunit-DINO	Baseline	0.990±0.006		0.964±0.015		0.963±0.015		0.955±0.027	
	LSO-Center	0.985±0.016	−0.50%	0.969±0.029	+0.50%	0.968±0.030	+0.50%	0.950±0.041	−0.50%
	LSO-Scanner	0.989±0.010	−0.10%	0.958±0.023	−0.60%	0.957±0.024	−0.60%	0.933±0.033	−2.20%
Phikon-v2	Baseline	0.995±0.004		0.968±0.017		0.967±0.020		0.955±0.043	
	LSO-Center	0.993±0.006	−0.20%	0.967±0.019	−0.10%	0.967±0.020	0.00%	0.943±0.029	−1.20%
	LSO-Scanner	0.992±0.008	−0.30%	0.958±0.018	−1.00%	0.957±0.020	−1.00%	0.923±0.033	−3.20%
Hibou-L	Baseline	0.997±0.002		0.966±0.015		0.965±0.016		0.955±0.036	
	LSO-Center	0.995±0.004	−0.20%	0.971±0.020	+0.50%	0.971±0.019	+0.60%	0.973±0.031	+1.80%
	LSO-Scanner	0.994±0.006	−0.30%	0.967±0.015	+0.10%	0.967±0.016	+0.20%	0.967±0.019	+1.20%
UNI2-h	Baseline	0.998±0.001		0.976±0.014		0.976±0.013		0.976±0.015	
	LSO-Center	0.997±0.003	−0.10%	0.975±0.021	−0.10%	0.975±0.021	−0.10%	0.965±0.030	−1.10%
	LSO-Scanner	0.995±0.005	−0.30%	0.968±0.015	−0.80%	0.968±0.015	−0.80%	0.957±0.017	−1.90%
Virchow2-CLS	Baseline	0.999±0.001		0.978±0.015		0.978±0.015		0.980±0.012	
	LSO-Center	0.999±0.001	0.00%	0.980±0.016	+0.20%	0.980±0.016	+0.20%	0.973±0.022	−0.70%
	LSO-Scanner	0.996±0.005	−0.30%	0.980±0.011	+0.20%	0.980±0.011	+0.20%	0.970±0.022	−1.00%
H-optimus-0	Baseline	0.997±0.002		0.974±0.014		0.974±0.014		0.972±0.021	
	LSO-Center	0.993±0.007	−0.40%	0.973±0.024	−0.10%	0.972±0.024	−0.20%	0.960±0.032	−1.20%
	LSO-Scanner	0.994±0.006	−0.30%	0.962±0.021	−1.20%	0.961±0.022	−1.30%	0.940±0.028	−3.20%
Prov-GigaPath	Baseline	0.998±0.002		0.976±0.016		0.976±0.016		0.976±0.015	
	LSO-Center	0.994±0.006	−0.40%	0.968±0.028	−0.80%	0.967±0.028	−0.90%	0.955±0.038	−2.10%
	LSO-Scanner	0.988±0.013	−1.00%	0.958±0.029	−1.80%	0.958±0.030	−1.80%	0.940±0.033	−3.60%
Lunit-BT	Baseline	0.991±0.007		0.958±0.030		0.955±0.035		0.938±0.065	
	LSO-Center	0.984±0.018	−0.70%	0.958±0.029	0.00%	0.957±0.029	+0.20%	0.945±0.036	+0.70%
	LSO-Scanner	0.986±0.013	−0.50%	0.958±0.029	0.00%	0.958±0.030	+0.30%	0.940±0.033	+0.20%
CTransPath	Baseline	0.993±0.007		0.968±0.026		0.966±0.030		0.947±0.059	
	LSO-Center	0.988±0.014	−0.50%	0.962±0.024	−0.60%	0.961±0.025	−0.50%	0.935±0.038	−1.20%
	LSO-Scanner	0.991±0.009	−0.20%	0.953±0.021	−1.50%	0.953±0.022	−1.30%	0.913±0.037	−3.40%

**Table 6:** Overall performance and class-wise F1-scores on BEETLE for four-class patch-level tissue classification, evaluated under the Baseline 5-fold CV protocol and the source-aware protocols LSO-Center and LSO-Scanner. Values are reported as mean ± standard deviation.

Model	Setting	Overall				Class-Wise F1-Score			
		Accuracy	F1-Score	AUROC	Sensitivity	Other	Non-Invasive	Invasive	Necrosis
CONCH	Baseline	0.876±0.023	0.834±0.025	0.981±0.005	0.879±0.029	0.923±0.016	0.872±0.027	0.805±0.065	0.735±0.063
	LSO-Center	0.829±0.046	0.749±0.063	0.962±0.013	0.821±0.029	0.896±0.048	0.611±0.180	0.725±0.149	0.764±0.065
	LSO-Scanner	0.814±0.075	0.691±0.129	0.960±0.019	0.790±0.067	0.892±0.054	0.590±0.200	0.603±0.279	0.680±0.262
Lunit-DINO	Baseline	0.866±0.017	0.818±0.021	0.978±0.005	0.859±0.044	0.926±0.013	0.846±0.028	0.780±0.058	0.722±0.062
	LSO-Center	0.787±0.050	0.704±0.050	0.951±0.016	0.781±0.021	0.892±0.044	0.489±0.166	0.671±0.154	0.762±0.079
	LSO-Scanner	0.775±0.077	0.651±0.108	0.952±0.019	0.755±0.043	0.884±0.057	0.484±0.195	0.558±0.258	0.678±0.255
Phikon-v2	Baseline	0.872±0.027	0.825±0.033	0.978±0.009	0.860±0.053	0.924±0.013	0.857±0.029	0.792±0.078	0.728±0.059
	LSO-Center	0.785±0.043	0.685±0.041	0.939±0.016	0.739±0.028	0.882±0.038	0.488±0.178	0.656±0.147	0.714±0.082
	LSO-Scanner	0.767±0.087	0.635±0.107	0.940±0.022	0.716±0.039	0.867±0.067	0.479±0.187	0.542±0.258	0.652±0.260
Hibou-L	Baseline	0.893±0.027	0.854±0.035	0.983±0.008	0.884±0.048	0.933±0.018	0.890±0.025	0.833±0.059	0.761±0.075
	LSO-Center	0.805±0.038	0.723±0.052	0.953±0.017	0.780±0.028	0.881±0.040	0.559±0.176	0.679±0.157	0.775±0.113
	LSO-Scanner	0.784±0.071	0.659±0.112	0.951±0.021	0.756±0.042	0.873±0.055	0.537±0.190	0.554±0.263	0.673±0.258
UNI2-h	Baseline	0.893±0.030	0.859±0.039	0.983±0.008	0.883±0.046	0.934±0.015	0.891±0.041	0.819±0.062	0.790±0.064
	LSO-Center	0.857±0.038	0.780±0.051	0.967±0.013	0.820±0.029	0.903±0.035	0.674±0.195	0.776±0.112	0.767±0.058
	LSO-Scanner	0.837±0.063	0.714±0.119	0.964±0.017	0.795±0.056	0.897±0.043	0.647±0.197	0.634±0.283	0.679±0.230
Virchow2-CLS	Baseline	**0.903 ** ±0.027	0.869±0.037	0.986±0.006	0.895±0.043	0.937±0.020	0.910±0.031	0.842±0.055	0.789±0.067
	LSO-Center	0.865±0.042	0.800±0.061	0.968±0.013	0.844±0.033	0.905±0.039	0.724±0.175	0.784±0.108	0.787±0.076
	LSO-Scanner	0.847±0.057	0.733±0.126	0.969±0.014	0.807±0.070	0.900±0.040	0.678±0.188	0.656±0.285	0.700±0.260
H-optimus-0	Baseline	0.900±0.026	0.865±0.033	0.986±0.006	0.893±0.045	0.934±0.020	0.910±0.027	0.841±0.055	0.778±0.064
	LSO-Center	0.851±0.033	0.777±0.044	0.965±0.013	0.818±0.026	0.899±0.036	0.672±0.165	0.762±0.121	0.773±0.095
	LSO-Scanner	0.839±0.054	0.717±0.113	0.965±0.013	0.789±0.050	0.897±0.043	0.652±0.162	0.639±0.281	0.681±0.259
Prov-GigaPath	Baseline	0.885±0.023	0.844±0.029	0.982±0.005	0.872±0.043	0.930±0.015	0.878±0.021	0.813±0.069	0.753±0.059
	LSO-Center	0.826±0.041	0.732±0.056	0.957±0.018	0.779±0.028	0.894±0.037	0.596±0.207	0.714±0.174	0.725±0.058
	LSO-Scanner	0.800±0.076	0.674±0.114	0.955±0.018	0.761±0.053	0.876±0.067	0.577±0.196	0.590±0.284	0.653±0.238
Lunit-BT	Baseline	0.844±0.019	0.789±0.020	0.970±0.007	0.838±0.040	0.912±0.018	0.816±0.020	0.749±0.064	0.678±0.061
	LSO-Center	0.783±0.034	0.689±0.036	0.944±0.015	0.768±0.043	0.880±0.037	0.488±0.203	0.674±0.141	0.713±0.094
	LSO-Scanner	0.782±0.051	0.649±0.097	0.948±0.016	0.753±0.055	0.881±0.043	0.493±0.202	0.572±0.265	0.650±0.243
CTransPath	Baseline	0.874±0.020	0.826±0.023	0.979±0.007	0.869±0.039	0.925±0.016	0.860±0.020	0.808±0.055	0.713±0.058
	LSO-Center	0.803±0.040	0.698±0.056	0.946±0.014	0.768±0.053	0.891±0.037	0.513±0.172	0.676±0.201	0.712±0.073
	LSO-Scanner	0.791±0.075	0.654±0.108	0.949±0.021	0.747±0.064	0.882±0.063	0.509±0.185	0.572±0.289	0.653±0.251

**Table 7:** BEETLE performance gaps (%) relative to the Baseline for each FM under LSO-Center and LSO-Scanner, reported for overall metrics and class-wise F1-scores.

Model	Overall (%)								Class-Wise F1-Score (%)							
	Accuracy		F1-Score		AUROC		Sensitivity		Other		Non-Invasive		Invasive		Necrosis	
	$\Delta_{\mathrm{Center}}$	$\Delta_{\mathrm{Scanner}}$	$\Delta_{\mathrm{Center}}$	$\Delta_{\mathrm{Scanner}}$	$\Delta_{\mathrm{Center}}$	$\Delta_{\mathrm{Scanner}}$	$\Delta_{\mathrm{Center}}$	$\Delta_{\mathrm{Scanner}}$	$\Delta_{\mathrm{Center}}$	$\Delta_{\mathrm{Scanner}}$	$\Delta_{\mathrm{Center}}$	$\Delta_{\mathrm{Scanner}}$	$\Delta_{\mathrm{Center}}$	$\Delta_{\mathrm{Scanner}}$	$\Delta_{\mathrm{Center}}$	$\Delta_{\mathrm{Scanner}}$
CONCH	−4.65	−6.14	−8.47	−14.24	−1.98	−2.09	−5.83	−8.91	−2.63	−3.05	−26.12	−28.18	−8.04	−20.23	+2.91	−5.51
Lunit-DINO	−7.82	−9.06	−11.49	−16.74	−2.74	−2.59	−7.75	−10.38	−3.36	−4.16	−35.70	−36.24	−10.91	−22.13	+4.03	−4.42
Phikon-v2	−8.68	−10.51	−14.02	−19.01	−3.81	−3.72	−12.06	−14.41	−4.21	−5.70	−36.91	−37.83	−13.57	−24.94	−1.42	−7.59
Hibou-L	−8.78	−10.86	−13.09	−19.53	−3.01	−3.23	−10.35	−12.84	−5.18	−6.00	−33.14	−35.35	−15.35	−27.90	+1.33	−8.88
UNI2-h	−3.64	−5.64	−7.85	−14.45	−1.64	−1.90	−6.29	−8.75	−3.12	−3.71	−21.65	−24.40	−4.36	−18.58	−2.26	−11.10
Virchow2-CLS	−3.83	−5.59	−6.92	−13.59	−1.77	−1.74	−5.13	−8.82	−3.20	−3.71	−18.59	−23.21	−5.74	−18.58	−0.16	−8.90
H-optimus-0	−4.92	−6.12	−8.89	−14.80	−2.07	−2.04	−7.45	−10.38	−3.44	−3.66	−23.72	−25.73	−7.92	−20.22	−0.47	−9.60
Prov-GigaPath	−5.93	−8.49	−11.12	−16.98	−2.53	−2.68	−9.28	−11.14	−3.55	−5.40	−28.23	−30.14	−9.89	−22.31	−2.78	−10.04
Lunit-BT	−6.05	−6.16	−10.01	−13.96	−2.56	−2.17	−7.01	−8.53	−3.22	−3.08	−32.84	−32.31	−7.47	−17.67	+3.47	−2.79
CTransPath	−7.15	−8.33	−12.85	−17.22	−3.35	−3.05	−10.10	−12.13	−3.39	−4.28	−34.69	−35.02	−13.16	−23.53	−0.18	−6.06

## Data Availability

The data presented in this study were derived from the following resources available in the public domain: BEETLE (https://zenodo.org/records/16812932, accessed on 5 August 2026) and SEMICOL (https://www.semicol.org/data/, accessed on 5 August 2026).

## Abbreviations

The following abbreviations are used in this manuscript:

WSI	Whole-Slide Image
H&E	Hematoxylin and Eosin
SSL	Self-Supervised Learning
TCGA	The Cancer Genome Atlas
JB	Jules Bordet Institute
NKI	Netherlands Cancer Institute
SCH	Santa Chiara Hospital
RUMC	Radboud University Medical Center
ViT	Vision Transformer
FM	Foundation Model
MI	Multiple Instance
MLP	Multi-Layer Perceptron
AUROC	Area Under the ROC Curve
CV	Cross-Validation
LSO	leave-source-out
LSO-Center	leave-center-out
LSO-Scanner	leave-scanner-out
CPath	Computational Pathology
NZ	NanoZoomer
P	Pannoramic
